# The respiratory research agenda in primary care in Portugal: a Delphi study

**DOI:** 10.1186/s12875-016-0512-1

**Published:** 2016-08-31

**Authors:** Vera Araújo, Pedro M. Teixeira, John Yaphe, Jaime Correia de Sousa

**Affiliations:** 1 School of Health Sciences, University of Minho, Braga, Portugal; 2 ICVS/3B’s - PT Government Associate Laboratory, Life and Health Sciences Research Institute (ICVS), School of Health Sciences, University of Minho, Campus de Gualtar, 4710-057 Braga, Portugal; 3 Horizonte Family Health Unit, Matosinhos, Porto Portugal

## Abstract

**Background:**

A research agenda can help to stimulate and guide research. The International Primary Care Respiratory Group (IPCRG) published a Research Needs Statement (RNS) in 2010 in which 145 research questions were identified. In 2012, priorities for respiratory research were established, based on these questions. To date, there has been no statement on primary care respiratory research needs in Portugal. The aim of the study was to develop a national consensus on research priorities in respiratory diseases in primary care in Portugal and to assess the applicability of the priorities for respiratory research set by the IPCRG.

**Method:**

We conducted a Delphi study by electronic mail with a panel of experts on respiratory disease from primary and secondary care in Portugal. In the first round, the research needs in respiratory disease in Portugal were identified. In the second round, 196 research questions in six disease areas, derived from the first round and from the IPCRG Respiratory needs statement, were prioritised on a five-point Likert-type scale. In the third round, the questions were prioritized again with feed-back provided on the median scores for each item in the second round. Consensus was considered to have been reached when 80 % of the participants gave a score of 4 or 5 out of five on a given item.

**Results:**

The 40 experts identified 121 respiratory research questions in Round 1 and expressed their views on 196 questions in Rounds 2 and 3. Twelve research questions (6 %) reached consensus. There were five questions in the asthma domain on early diagnosis, pulmonary function tests, the use of inhalers, and adherence to treatment. There were four questions in the chronic obstructive pulmonary disease domain on vaccinations, on routine monitoring and evaluation of treatment, on diagnosis, and on adherence to treatments. There was one question in the smoking domain on the effects of brief counselling. There were two questions on respiratory tract infections on the treatment of children and on the prescription of antibiotics. An additional 23 research questions (12 %) achieved consensus between 75 and 79 %.

**Conclusion:**

The results reflect the Portuguese reality in response the international agenda for research on respiratory diseases published by the IPCRG. They can support the development of future respiratory disease research in Portugal.

## Background

Respiratory diseases (RD) are a significant part of the Priority Agenda for the Prevention and Control of Noncommunicable Diseases published by the World Health Organization (WHO) in 2011 [1]. This document stressed the importance of adopting measures in Primary Care (PC) for the control and prevention of RD. Chronic and acute respiratory diseases are managed by health professionals in primary care. The World Organization of Family Doctors (WONCA) has expressed the need to adopt a critical approach to clinical practice that is research-based as a core competence of scientific expertise [2]. This is true for respiratory conditions in primary care as well.

The burden of respiratory disease is significant. Lower respiratory tract infections and neoplasms of the trachea, bronchus and lung were among the top ten causes of death worldwide in 2012 [3]. Chronic obstructive pulmonary disease (COPD), asthma, rhinitis, respiratory infections, and tobacco consumption are the main challenges in RD not only because of their prevalence but also due to their impact on health and quality of life [4–6].

The International Primary Care Respiratory Group (IPCRG) is a non-governmental organization composed of groups of professionals from 24 countries. It was created with the mission of sharing and disseminating scientific evidence about RD [7]. In 2010, the IPCRG published the Research Needs Statement (RNS) listing the research needs identified by professionals with experience and interest in RD in primary care [8]. In 2012 the IPCRG published a list of priorities for respiratory research needs for primary care [9]. A set of 145 research questions related to asthma, rhinitis, COPD, nicotine dependence, and respiratory infections were ranked by experts in RD, producing an international research agenda.

In Portugal, RD are the third leading causes of death. Portugal has the second highest mortality rate for RD in the European Union (EU) [3]. The mortality associated with pneumonia is twice the EU rate and the mortality from asthma and COPD are slightly below the EU average [10]. It is expected that RD in Portugal will continue to cause significant morbidity and mortality with a tendency to an increase in prevalence rates [10]. In Portugal, chronic respiratory diseases caused 22 deaths per 100,000 inhabitants in 2012 [11]. Research in this area represents a strategic axis in the National Programme for the Prevention and Control of Tobacco [12].

Asthma and COPD studies in Portugal have included surveys and a sentinel surveillance network [13–16] and indicated a prevalence of asthma ranging from 7.8 % (95 % CI: 7.0–8.8) [14] to 10.24 % (95 % CI: 8.16–12.32) [15] and an incidence rate of 2.02/1000/year (95 % CI: 1.8–2.2) in the period from 2007 to 2009 [16]. The prevalence of COPD in adults, aged 40 years or older has been estimated in different surveys ranging from 9 to 14.2 % [17, 18]. The COPD incidence rate has also been established in 1,62/1000/year (95 % CI: 1,39–1,87/1000) in the period from 2007 to 2009 [16].

The National Programme for Respiratory Diseases is one of the priority programs of the National Health Plan 2012–2016 [10]. In 2013, the Research Agenda in the National Health Plan and National Programmes Priority was published. This document produced a short list of broad research needs in chronic respiratory diseases that aim to improve knowledge of RD in Portugal [19, 20]. It is important to study research needs in this area and to set a schedule for the development of research that provides evidence for clinical practice that is adapted to the Portuguese context. This study aimed to develop a national research consensus in respiratory diseases in primary care in Portugal and to assess the applicability of the priorities for respiratory research needs in Primary Health Care set by the IPCRG.

## Method

The study was designed using the Delphi method. The ethics subcommittee for Life and Health Sciences at the University of Minho assessed and approved the research protocol.

### Delphi method

The Delphi method is used in health services research and medicine to obtain expert consensus [21]. It consists of a series of rounds of data collection designed to reach consensus among a group of experts in a given area. Questions are sent to the participants by post or electronic means allowing for greater geographic coverage and avoiding the potential bias arising in face-to-face meetings [22]. Anonymity of responses is another important feature, minimizing the effect of leadership bias [23].

This method requires the successive application of questions including feedback of results from previous rounds or ‘iteration with return process’ [22]. In subsequent rounds, participants have access to the results of the group obtained in the previous round.

### Selection of participants

The appropriate selection of the expert panel is a key step because it is closely related to the quality of the results [21–24]. Some important eligibility criteria are experience, knowledge and interest in the area in question, the ability to contribute to research, and the ability to review initial opinions to achieve a group consensus. There is no ideal number of participants in a Delphi study. Most studies include 15–20 participants [22].

The panel of participants in this study was composed of experts in RD with clinical experience and scientific knowledge in this area. The purpose was to recruit a number of geographically diverse individuals, including experts in Family Medicine, Pulmonology, Allergology, and other experts from the clinical area and research in RD. The research team invited 79 experts to participate in the study.

### Procedure

The Delphi process was held from September to November 2014. The procedure was carried out online by e-mail and using the Google Drive® forms and an Excel® spreadsheet. Communication with participants used an electronic mail address created for this purpose with restricted access to researchers. All the messages sent and the data collection procedures were checked by the researchers to assure clarity, simplicity, and functionality of the process. To ensure confidentiality and anonymity, all participants were assigned a personal identification code that was used throughout the study. An initial invitation was sent to all potential participants (*N* = 79). This included a brief description of the study and its objectives. The message contained a link to a reply form that contained a consent form and a request for socio-demographic data, including the age, gender, and profession of the participant.

The final panel included 40 of the 79 experts previously contacted, for a response rate of 50.6 %. The panel was composed by 24 males (60 %) and 16 females (40 %) with an age range between 26 and 62 years and a mean age of 43 years. Table 1 shows the characteristics of the expert panel by geographic distribution and profession. Participants came from seven districts including Aveiro, Braga, Coimbra, Lisbon, Porto, Santarém, and Faro. The panel included 39 physicians (98 %) and 1 RD consultant in clinical practice and research (2 %). There were 31 specialists in Family Medicine (79 %), 2 in pulmonology (5 %), 2 in immunoallergology (5 %), and 3 trainees in Family Medicine (8 %). In addition to clinical activity, 11 participants (22 %) also had academic duties in undergraduate and postgraduate training.

**Table 1:** Location and specialty of Delphi respiratory research panel participants (*n* = 40)

District	*n*	Percentage	Specialty
Aveiro	3	8 %	Family Medicine.
Braga	8	20 %	Family Medicine (7), 1 Family Medicine trainee (1)
Coimbra	2	5 %	Family Medicine
Lisboa	4	10 %	Family Medicine (2),Pulmonology (1) Research Consultant (1)
Porto	21	53 %	Family Medicine (16) Family Medicine trainee (1) Imunoalergology (2) Pulmonology (1)
Santarém	1	3 %	Family Medicine
Faro	1	3 %	Family Medicine
Total	40	100 %	

#### Analysis

##### Round 1

The first round began with an open question: “*What research needs do you identify regarding respiratory diseases in Primary Care in Portugal, for chronic obstructive pulmonary disease, asthma, rhinitis, nicotine dependence, and respiratory infections?*” After receiving the answers from round 1, the research team designed a questionnaire that combined the needs identified by the experts in Portugal and the research questions in the Research Needs Statement of the IPCRG. Research questions were divided into six areas: general questions, asthma, rhinitis, COPD, smoking, and respiratory infections. Within each domain there was a division into subcategories.

##### Round 2

In the second round, the questionnaire was sent to the panel members who participated in Round 1. The email contained a link to access an online version of the questionnaire with instructions and an Excel spread sheet file for download, completion, and return. Each research topic was to be rated by the participants on a five-point Likert-type scale, with 1 corresponding to the lowest priority and 5 the highest priority for research. Experts were asked to rate each research topic focusing on its importance and feasibility of implementation in the Portuguese primary care context. After receiving all the responses in round 2, the researchers calculated the frequencies and the median value of the ratings given for each research topic.

##### Round 3

In the third round, each member of the panel received the questionnaire with feed-back including the median value of the group ratings in Round 2 and the participant’s own previous answers. All members were asked to complete the questionnaire for a second time. In this round, each participant was asked to reflect and review the previous answers, given the feedback information, in order to reach a consensus.

## Results

Consensus was reached on the research topics that obtained an agreement of 80 % for a score of 4 or 5 out of 5. All 40 experts who initially consented to join the study participated in Round 1 (100 %). In Round 2 there were 32 replies received (80 % of the original panel) and in Round 3 there were 28 replies received (70 % of the original panel). The dropout rate of the Delphi study was considered acceptable, given the characteristics of the method [21–24].

### Round 1

In the first round, 121 research topics were identified by the panel. The research team found that of these, 44 overlapped with topics on the Research Needs Agenda of the IPCRG and 77 were new topics (ie non-overlapping with the RNS). The research topics identified covered the domains of asthma (4 topics), rhinitis (5 topics), COPD (19 topics), smoking (17 topics), and respiratory infections (16 topics). There were 16 topics identified that did not fit in any of these areas. They were classified in a new domain called “general respiratory diseases”.

### Round 2

The new questionnaire developed for rounds 2 and 3 included 196 topics. Of these, 77 (39.3 %) were new topics suggested by the participants in Round 1, 75 topics (38.3 %) that were taken from the Respiratory Needs Statement of the IPCRG, and 44 topics (22.4 %) that were common to Round 1 suggestions and the RNS.

The distribution of the topics by the six domains is presented in Table 2. There were 46 topics (23.5 %) in the COPD domain, 45 (23.0 %) in the asthma domain, 33 (16.8 %) in the respiratory infections domain, 30 (15.3 %) in the smoking domain, 26 (13.3 %) in the rhinitis domain, and 16 topics (8.2 %) in the general respiratory diseases domain.

**Table 2:** Respiratory research topics by source, domain and category

Disease Domain and Category	Topic Source			Total
	Portugal	RNS/Portugal	RNS	196
General Respiratory Disease	16	0	0	16
Evaluation	4	0	0	4
Diagnosis	1	0	0	1
Continuous Professional Development	1	0	0	1
Epidemiology	2	0	0	2
Comorbidity	1	0	0	1
Practice organization	4	0	0	4
Treatment	3	0	0	3
Asthma	4	18	23	45
Self-treatment	1	1	4	6
Evaluation	1	7	1	9
Comorbidity	0	1	1	2
Compliance	1	1	0	2
Diagnosis	1	4	4	9
Diversity	0	0	2	2
Continuous Professional Development	0	1	0	1
Pharmacological	0	0	1	1
Practice organization	0	1	3	4
Laboratory tests	0	0	1	1
Treatment	0	2	6	8
Rhinitis	5	6	15	26
Self-treatment	0	0	4	4
Evaluation	0	1	2	3
Comorbidity	1	0	1	2
Compliance	0	0	1	1
Development	1	1	1	3
Diagnosis	0	1	1	2
Epidemiology	2	0	0	2
Pharmacology	0	0	2	2
Prevention	0	1	0	1
Laboratory tests	0	0	1	1
Treatment	1	2	2	5
COPD	19	10	17	46
Self-treatment	1	1	0	2
Evaluation	6	2	1	9
Comorbidity	1	1	1	3
Diagnosis	2	3	2	7
Continuous Professional Development	3	1	1	5
Pharmacology	0	0	1	1
Practice organization	4	0	7	11
Prevention	1	0	2	3
Laboratory tests	0	0	1	1
Treatment	1	2	1	4
Smoking	17	5	8	30
Evaluation	5	3	0	8
Compliance	0	0	1	1
Continuous Professional Development	3	1	1	5
Pharmacology	1	0	1	2
Practice organization	4	1	1	6
Prevention	3	0	1	4
Treatment	1	0	3	4
Respiratory Infections	16	5	12	33
Self-treatment	0	1	0	1
Evaluation	1	2	0	3
Diagnosis	2	0	0	2
Diversity	1	0	0	1
Continuous Professional Development	1	0	0	1
Epidemiology	2	0	0	2
Pharmacology	0	0	2	2
Practice organization	1	0	2	3
Prevention	1	0	3	4
Laboratory tests	0	1	1	2
Treatment	7	1	4	12

The topics were distributed in 14 subcategories: 36 (18.4 %) in literature review, 36 (18.4 %) in treatment, 28 (14.3 %) in practice organization, 21 (10.7 %) in diagnosis, 13 (6.6 %) in self-care, 13 (6.6 %) in continuing professional development, 12 (6, 1 %) in prevention, 8 (4.1 %) in comorbidity, 8 (4.1 %) in medications, 6 (3.1 %) in epidemiology, 5 (2, 6 %) in laboratory tests, 4 (2 %) in compliance, 3 (1.5 %) in development, and 3 (1.5 %) in diversity.

### Round 3

Of the 196 topics in the questionnaire, 12 (6 %) reached the level of 80 % consensus with a score of 4 or 5 out of 5. The consensus ranged between 82 and 89 %. Median values remained unchanged from Round 2 to Round 3.

The topics identified by category and domains are shown in Table 3. The 12 research topics that obtained consensus were distributed in four domains: 5 on asthma (42 %), 4 on COPD (33 %), 1 on smoking (8 %) and 2 on respiratory infections (17 %). These topics were common to the Portuguese experts and the RNS in 5 cases (42 %). 4 (33 %) of these were suggested by the Portuguese experts in round 1 and 3 (25 %) were from the RNS. Research topics were identified in the categories of treatment (6 topics), literature review (2 topics), diagnosis (2 topics), compliance (1 topic) and prevention (1 topic).

**Table 3:** Consensus on respiratory disease research priorities in Portugal

Domain	Category (%)	Source	Research Topic and % agreement	
Asthma	Compliance (*n* = 1; 8.3 %)	RNS/PT	1. What is the degree of adherence to therapy among Portuguese asthma patients? 2. How can the adherence problems be resolved (especially in subgroups such as adolescent patients with asthma)?	89 %
COPD	Prevention (*n* = 1; 8.3 %)	PT	3. How important are the anti-influenza and anti-pneumococcal vaccination in COPD? 4. Are our patients vaccinated appropriately with the anti-pneumococcal vaccine? 5. What can we do to improve this?	89 %
COPD	Treatment (*n* = 6; 50 %)	PT	6. What is the degree of adherence to maintenance treatments in COPD?	89 %
Smoking	Treatment (*n* = 6; 50 %)	RNS	7. How may short counselling be used more effectively to increase motivation to quit smoking? 8. What methods are efficient for the busy family doctor?	89 %
Asthma	Evaluation (*n* = 2; 16.6 %)	RNS/PT	9. What is the role of lung function tests in regular monitoring of asthma patients in primary care? 10. What is the appropriate frequency of tesing for each degree of severity and level of control?	86 %
Asthma	Diagnosis (*n* = 2; 16.6 %)	RNS/PT	11. How can you diagnose asthma earlier in Primary Care? 12. What causes under-diagnosis?	82 %
Asthma	Treatment (*n* = 6; 50 %)	RNS/PT	13. How empowered are asthma patients? 14. What are their preferences in usage of inhalers? 15. How can you identify good and poor inhaler technique? 16. What is the best strategy to ensure good inhaler technique?	82 %
Asthma	Treatment (*n* = 6; 50 %)	RNS	17. What is the most cost-effective approach for inhaler use?	82 %
COPD	Evaluation (*n* = 2; 16.6 %)	PT	18. What measurements (spirometry, dyspnea scores, exercise tolerance, symptom scores, control scores, specific COPD questionnaires or generic Quality of Life scores) are viable and provide useful information for routine monitoring and evaluation of the effectiveness of treatment in primary care? 19. What is the ideal frequency of respiratory function tests for monitoring?	82 %
COPD	Diagnosis (*n* = 2; 16.6 %)	RNS/PT	20. What is the best way to diagnose COPD in primary care?	82 %
Respiratory Infections	Treatment (*n* = 6; 50 %)	RNS	21. Can delayed prescribing reduce the use of antibiotics without compromising the results?	82 %
Respiratory Infections	Treatment (*n* = 6; 50 %)	PT	22. What is the best strategy to approach airway infections in children in Primary Care? 23. Are children with the upper respiratory tract infections being treated excessively with antibiotics?	82 %

*PT* Portuguese, *RNS* research needs statement

The 12 topics that achieved consensus were from four distinct domains. Five were on asthma, including two questions on early diagnosis, evaluation, pulmonary function tests, treatment (the use of inhalers), and adherence to therapy. Four were on COPD including questions on prevention of exacerbations using vaccination, measurements for routine monitoring and evaluation of treatment, diagnosis in primary care, and adherence to maintenance treatments. One question related to the effectiveness of brief counselling for smoking cessation in primary care. Two questions related to respiratory Infections, regarding the treatment of children and the prescription of antibiotics.

There were 23 topics (12 %) that almost reached consensus (75–79 % agreement). Among these topics there were 16 (69 %) topics identified exclusively by the Portuguese experts, 5 (22 %) that were common to the Research Needs Statement and the Portuguese panel and 2 topics (9 %) that came from RNS. Regarding the disease domains, 8 (35 %) were on COPD, 5 (22 %) on asthma, 4 (17 %) on general respiratory diseases, 4 on respiratory infections and 2 (9 %) on smoking. Regarding the distribution of the questions by categories, 5 (22 %) referred to diagnosis, 4 (17 %) to literature review, 4 (17 %) to treatment, 3 (13 %) to self-care, 2 (9 %) to epidemiology, 2 (9 %) to prevention and 1 (4 %) question each related to compliance, continuous professional development and practice organization.

## Discussion and conclusion

This Delphi study of research needs in respiratory diseases in Portugal produced a list of 121 research questions in the first round. Many of these topics (77; 63.6 %) did not appear in the Research Needs Statement (RNS) published by the IPCRG. This suggests that these questions are more appropriate for Portugal. Of the 12 topics that achieved consensus among the experts in subsequent rounds, seven also appear in the IPCRG list. One question has not achieved consensus in the IPCRG agenda and four were unique to Portugal and do not appear in the RNS of the IPCRG.

There are other differences between this study and the RNS. The domains with the highest number of topics suggested in this study were COPD, asthma, and respiratory infections. The top three domains in the RNS were asthma, COPD, and rhinitis. The sub-categories of literature review, treatment, and practice organization contained the greatest number of research questions in this study compared to the IPCRG study, which favoured diagnosis, literature review, and treatment.

The number of questions that reached consensus in this study (12) was lower than that obtained by IPCRG study (62 issues).

A large number of research questions (23 questions) achieved a borderline level of consensus (between 75 and 79 %). Some of these issues might have achieved consensus if additional rounds were held. These topics may be considered in future studies.

In 2013, the Directorate-General of Health (DGS) published a Research Agenda in the National Health Plan and National Programmes Priority, which includes items on research needs in respiratory diseases [10]. The DGS agenda has five items on respiratory diseases, none of them on asthma, allergic rhinitis or COPD, and 23 items on tobacco cessation, tobacco control and the empowerment of health professionals for tobacco control. When compared with the research needs identified in this study, the DGS agenda is more general, more focused on health policies, and does not provide guidance for clinical researchers.

Another research agenda in PHC, the Research Agenda for General Practice/Family Medicine and Primary Health Care in Europe, was prepared by the European General Practice Research Network EGPRN [25]. The methods used differ from the ones in the present study and the EGPRN agenda is more general and more focused on the WONCA definition of family medicine, without any specific items on respiratory problems, so comparisons are difficult. The UK Respiratory Research Collaborative (UKRRC) joined several organisations as an attempt to raise the profile of respiratory research in the UK, through an e-Delphi exercise. Though not specifically aimed at PHC, the conclusions of the study identified research needs similar to those found in the present study [26].

In 2011, the WHO published its prioritized research agenda for prevention and control of non-communicable diseases. There are some common points with our agenda, such as research on the prevalence of respiratory diseases, on disease burden, on risk factors and on comorbidities. Other common items include assessing gaps in access and affordability of essential technologies and medicines [1].

This study may contribute to the development of a Portuguese agenda for research in respiratory diseases. Priorities were defined for asthma, COPD, respiratory infections and smoking, with a strong focus on treatment. The unique nature of these questions, distinct from other published respiratory research agendas, suggests that this list is appropriate for Portugal.

Future studies will help to clarify the relevance and priorities of this list for patients, clinicians, researchers, funding bodies, and other stakeholders. It may be valuable to consider the topics that have obtained consensus among 75–79 % of participants in this study. The top 35 research questions explored here may form the Portuguese respiratory research agenda. This may help to guide future investment in research in respiratory diseases in Portugal.

## Abbreviations

COPD: Chronic obstructive pulmonary disease
EU: European Union
IPCRG: International Primary Care Respiratory Group
PHC: Primary health care
RD: Respiratory diseases
RNS: Research needs statement
WHO: World Health Organization
WONCA: World Organization of Family Doctors
